# Acid sphingomyelinase expression is associated with survival in resectable pancreatic ductal adenocarcinoma

**DOI:** 10.1007/s00109-023-02331-6

**Published:** 2023-05-29

**Authors:** Gregory C. Wilson, Sameer H. Patel, Jiang Wang, Kui Xu, Kevin M. Turner, Katrin Anne Becker, Alexander Carpinteiro, Ildiko Szabo, Syed A. Ahmad, Erich Gulbins

**Affiliations:** 1 Division of Surgical Oncology, Department of Surgery, University of Cincinnati College of Medicine, 231 Albert Sabin Way ML 05, Cincinnati, OH 45267-0558 USA; 2 Departments of Pathology and Laboratory Medicine, University of Cincinnati College of Medicine, Cincinnati, OH USA; 3 Institute of Molecular Biology, University Hospital Essen, University of Duisburg-Essen, Hufelandstrasse 55, 45122 Essen, Germany; 4 Department of Biology and CNR Institute of Neurosciences, University of Padua, Padua, Italy

**Keywords:** Acid sphingomyelinase, Sphingolipids, Pancreas cancer, Progression

## Abstract

**Abstract:**

Pancreatic adenocarcinoma (PDAC) is one of the most common cancers worldwide. Unfortunately, the prognosis of PDAC is rather poor, and for instance, in the USA, over 47,000 people die because of pancreatic cancer annually. Here, we demonstrate that high expression of acid sphingomyelinase in PDAC strongly correlates with long-term survival of patients, as revealed by the analysis of two independent data sources. The positive effects of acid sphingomyelinase expression on long-term survival of PDAC patients were independent of patient demographics as well as tumor grade, lymph node involvement, perineural invasion, tumor stage, lymphovascular invasion, and adjuvant therapy. We also demonstrate that genetic deficiency or pharmacological inhibition of the acid sphingomyelinase promotes tumor growth in an orthotopic mouse model of PDAC. This is mirrored by a poorer pathologic response, as defined by the College of American Pathologists (CAP) score for pancreatic cancer, to neoadjuvant therapy of patients co-treated with functional inhibitors of the acid sphingomyelinase, in particular tricyclic antidepressants and selective serotonin reuptake inhibitors, in a retrospective analysis. Our data indicate expression of the acid sphingomyelinase in PDAC as a prognostic marker for tumor progression. They further suggest that the use of functional inhibitors of the acid sphingomyelinase, at least of tricyclic antidepressants and selective serotonin reuptake inhibitors in patients with PDAC, is contra-indicated. Finally, our data also suggest a potential novel treatment of PDAC patients with recombinant acid sphingomyelinase.

**Key messages:**

- Pancreatic ductal adenocarcinoma (PDAC) is a common tumor with poor prognosis.
- Expression of acid sphingomyelinase (ASM) determines outcome of PDAC.
- Genetic deficiency or pharmacologic inhibition of ASM promotes tumor growth in a mouse model.
- Inhibition of ASM during neoadjuvant treatment for PDAC correlates with worse pathology.
- ASM expression is a prognostic marker and potential target in PDAC.

## Introduction

Pancreatic cancer is on track to become the third leading cause of cancer-related death in the world by 2025 [1–3]. Despite decades of advancement and research into the multimodal care of pancreatic adenocarcinoma (PDAC), the outlook remains grim. Five-year overall survival for all patients with pancreatic cancer has only recently risen above 10% [1, 3]. Unfortunately, the majority of patients with PDAC present with metastatic disease at time of diagnosis. Only a minority of patients present with potentially resectable disease and even these patients with “localized disease” experience high rates of distant recurrence and poor overall survival [2, 4]. The current state of PDAC outcomes remains a humbling reminder that significant work remains in our understanding of the molecular basis of this disease and our ability to treat it. Much work is still needed in developing effective treatment strategies to incorporate local and systemic therapies in novel ways to treat the primary tumor as well as micrometastatic disease likely present at the time of diagnosis. It is also necessary to define prognostic factors that allow individual treatments and to characterize signaling mechanisms in the tumor cells and/or host cells that determine tumor progression.

The acid sphingomyelinase (EC 3.1.4.12, sphingomyelin phosphodiesterase 1 (SMPD1); optimal pH 5.0) is a glycoprotein that functions as a lysosomal hydrolase, catalyzing the degradation of sphingomyelin to phosphorylcholine and ceramide [5, 6]. The enzyme mainly localizes to lysosomes, but it is also present in acidic compartments on the cell surface, since lysosomes are constantly recycling to and exchanging with the plasma membrane [7, 8]. Ceramide generated by the acid sphingomyelinase is then further metabolized to sphingosine, which is phosphorylated to sphingosine-1-phosphate. Ceramide can be also phosphorylated to ceramide-1-phosphate or further metabolized to glycosylated sphingolipids. The acid sphingomyelinase, ceramide- and its derivatives have been implied by many studies to be very important in the pathogenesis and treatment of tumors with chemotherapy and irradiation, for the regulation of the immune response and tumor vasculogenesis [9–11]. We and others have shown that the acid sphingomyelinase and ceramide play an important role in receptor signaling and regulation of the innate immune response [7, 12, 13]. The signaling function of the acid sphingomyelinase seems to be mainly regulated by the activity of acid sphingomyelinase on the cell surface resulting in the formation of ceramide in the outer leaflet of the cell membrane [7]. The generation of ceramide molecules within the outer leaflet alters the biophysical properties of the plasma membrane, because the very hydrophobic ceramide molecules spontaneously associate with each other to form small ceramide-enriched membrane domains that fuse and form large, highly hydrophobic, tightly packed, gel-like ceramide-enriched membrane domains [12, 14, 15]. These large, distinct, ceramide-enriched membrane domains have been shown to be crucially involved in cellular stress responses, such as induction of cell death [7, 14]. However, ceramide is also able to bind directly to signaling molecules such as e.g. cathepsins, some PKC isoforms, phosphatase 2A or Lc3B [16–19].

Although it is well known that sphingolipids are very important in tumor biology, it is unknown whether expression of the acid sphingomyelinase in malignant tumors, in particular in PDAC has an impact on the prognosis and long-term survival of patients.

Here, we investigated in three independent patient cohorts and in pharmacological and genetic mouse models the role of the acid sphingomyelinase for the prognosis of pancreas cancer.

## Results

The acid sphingomyelinase-ceramide system has been implied in the pathophysiology of malignant tumors [9–11], but its role in pancreas cancer is unknown. To analyze whether expression of the acid sphingomyelinase plays a role in human pancreatic ductal adenocarcinoma (PDAC), we correlated the expression of the acid sphingomyelinase in tissue samples from patients that were diagnosed with resectable PDAC, but not yet treated with any chemotherapy or surgical intervention, with the long-term survival of these patients. The patients underwent a surgery-first approach, i.e., surgery was performed prior to any other treatment, and therefore, we were able to obtain tumor specimen for histology analysis prior to any chemotherapy. Clinical data including patient demographics, clinical, operative, and pathologic characteristics, as well as survival, was determined (Tables 1 and 2).

**Table 1:** Patient demographics—resectable pancreatic ductal adenocarcinoma undergoing surgery-first approach treatment. The table indicates the patients’ demographics of all patients included in the cohort analyzed at the University of Cincinnati. Continuous variables compared with Student’s *t*-test. Categorical variables were compared with Fisher’s exact and Pearson’s chi-squared tests

	**All patients**	**SMPD1 low**	**SMPD1 high**	***p*-value**
	23	69.6% (*n* = 16)	30.4% (*n* = 7)	
**Age, yrs (mean, SEM)**	69.3 + / − 1.5	69.4 + / − 1.8	69.1 + / − 3.2	0.92 (Student’s *t*-test)
**Sex**				1.0 (Fisher’s exact)
*** Male***	60.9% (*n* = 14)	62.5% (*n* = 10)	57.1% (*n* = 4)	
*** Female***	39.1% (*n* = 9)	37.5% (*n* = 6)	42.9% (*n* = 3)	
**Race**				0.22 (Pearson’s chi sq)
*** White***	87.0% (*n* = 20)	93.8% (*n* = 15)	71.4% (*n* = 5)	
*** Black***	8.7% (*n* = 2)	6.2% (*n* = 1)	14.3% (*n* = 1)	
*** Hispanic***	4.3% (*n* = 1)	0	14.3% (*n* = 1)	
**Resection**				0.2 (Pearson’s chi sq)
*** Pancreatico-duodenectomy***	69.6% (*n* = 16)	68.8% (*n* = 11)	71.4% (*n* = 5)	
*** Distal***	26.1% (*n* = 6)	25% (*n* = 4)	28.6% (*n* = 2)	
*** Total***	4.3% (*n* = 1)	6.2% (*n* = 1)	0

**Table 2:** Pathology data from the University of Medicine Medical Center. The table gives the pathology characteristics of the surgery-first PDAC patients analyzed in the present study. Categorical variables were compared with Fisher’s exact and Pearson’s chi-squared tests

	**SMPD1 low** **(*n*** = **16)**	**SMPD1 high** **(*n*** = **7)**	***p*-value**
	69.6%	30.4%	
**Tumor grade**			0.17 (Pearson’s chi sq)
** 1—Well differentiated**	6.2% (*n* = 1)	0	
** 2—Moderately differentiated**	75% (*n* = 12)	42.9% (*n* = 3)	
** 3—Poorly differentiated**	19.8% (*n* = 3)	57.1% (*n* = 4)	
**Lymph node Involvement**	93.8% (*n* = 15)	85.7% (*n* = 6)	0.53 (Fisher’s exact)
**Perineural invasion**	100% (*n* = 16)	85.7% (*n* = 6)	0.30 (Fisher’s exact)
**Lymphovascular invasion**	62.5% (*n* = 10)	42.9% (*n* = 3)	0.65 (Fisher’s exact)
**Stage**			0.43 (Pearson’s chi sq
** I**	6.3% (*n* = 1)	0	
** IIA**	0% (*n* = 0)	14.3% (*n* = 1)	
** IIB**	62.5% (*n* = 10)	57.1% (*n* = 4)	
** III**	31.2% (*n* = 5)	28.6% (*n* = 2)	
**Adjuvant therapy**			*p* = 0.78 (Pearson’s chi sq)
** Gemcitabine-based**	56.2% (*n* = 9)	71.4% (*n* = 5)	
** FOLFIRINOX**	18.8% (*n* = 3)	14.3% (*n* = 1)	
** None**	25% (*n* = 4)	14.3% (*n* = 1)	

**Fig. 1:**
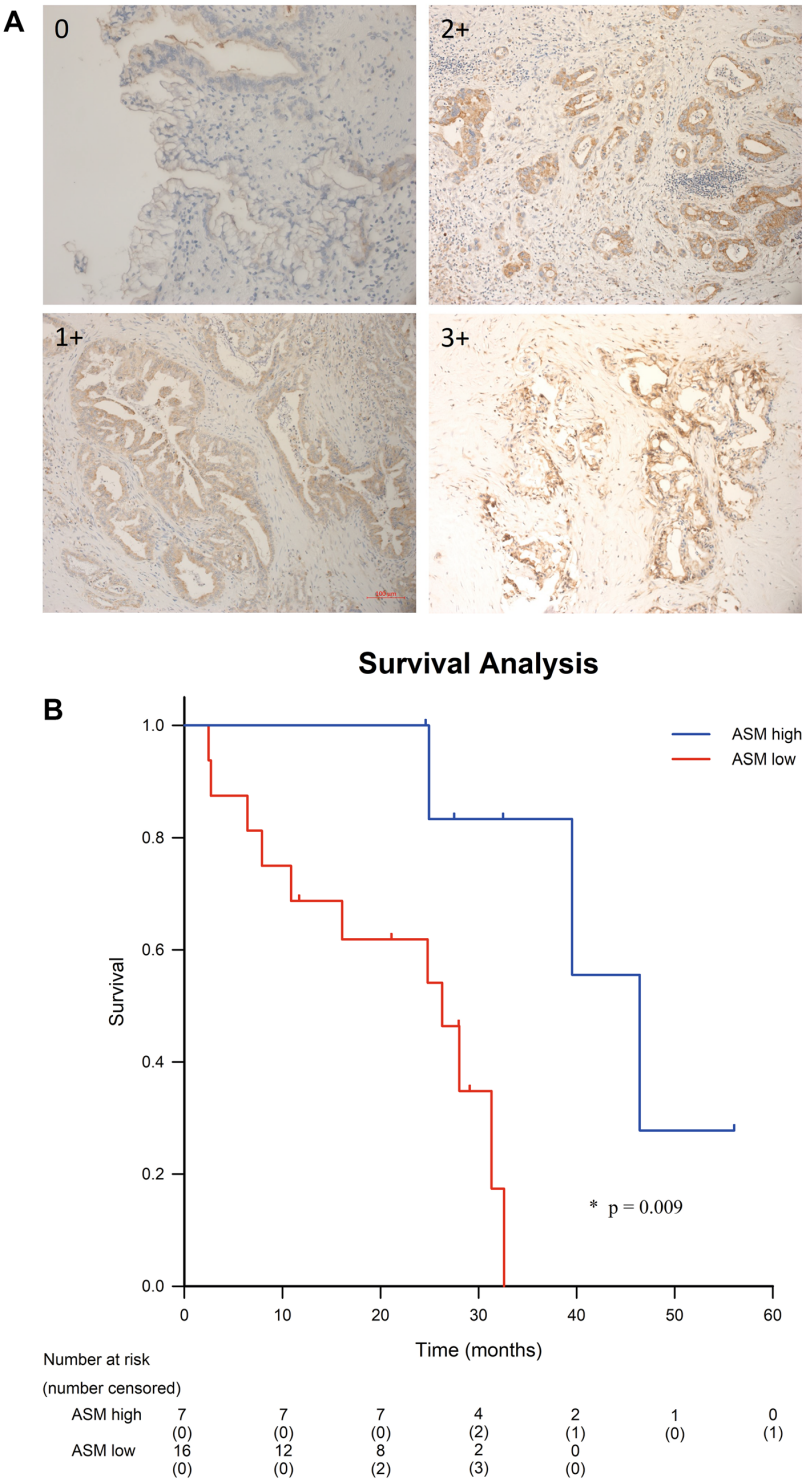
Acid sphingomyelinase expression correlates with survival in patients with pancreatic ductal adenocarcinoma. Shown are immunohistochemistry stainings of the acid sphingomyelinase (**A**) and overall survival in resectable pancreatic ductal adenocarcinoma (**B**). **A** IHC staining for acid sphingomyelinase was performed on human PDAC resection specimens (*n* = 23). All patients underwent a *surgery-first approach* with no preoperative therapy. Shown are representative examples from the 23 specimens. **B** Overall patient survival by ASM expression. Median overall survival was 26.3 months in the acid sphingomyelinase low cohort and 46.4 months in the acid sphingomyelinase high cohort. Low expression was defined as 0 or 1 + tumor staining and high expression as 2 + or 3 + staining. Overall survival was evaluated using the Kaplan–Meier estimator, for statistical analysis the log-rank test was used

The immunohistochemistry analysis of acid sphingomyelinase expression was graded from 0 (no detectable acid sphingomyelinase expression) to 3 + (strong expression of the acid sphingomyelinase) as shown in Fig. 1A. Correlation of the acid sphingomyelinase expression with patient survival data indicates that high expression of acid sphingomyelinase in the tumor tissue strongly correlated with improved prognosis of the patients with overall survival (Fig. 1B). Tumors with 2 + or 3 + staining for SMPD1 were categorized as SMPD1 high expression while those with 0 or 1 + staining were categorized at SMPD1 low expression.

Age, sex and race, the type of resection, tumor grade, lymph node involvement, perineural invasion, tumor stage, lymphovascular invasion, and adjuvant therapy did not differ between the groups with high and low expression of the acid sphingomyelinase (Tables 1 and 2).

The analysis revealed that a high expression of the acid sphingomyelinase strongly correlated with the prognosis and overall survival of the patients.

**Fig. 2:**
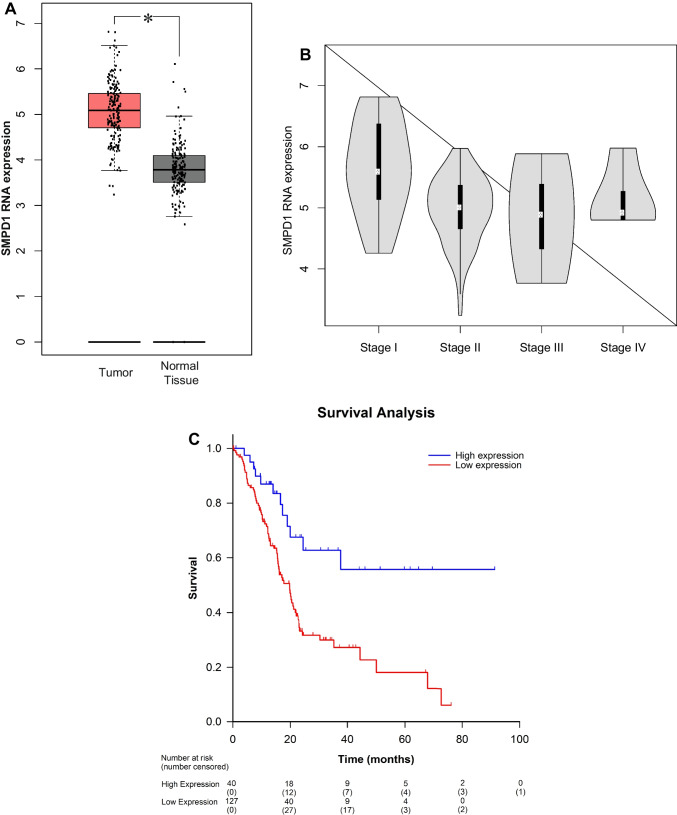
Acid sphingomyelinase expression in human pancreatic ductal adenocarcinoma. Shown are acid sphingomyelinase (SMPD1 is the gene symbol for the acid sphingomyelinase) expression levels in pancreatic adenocarcinoma (compared to normal pancreatic tissue and by cancer stage). **A** The mRNA expression of SMPD1 was assessed comparing tumor (red) and normal tissue (gray) from TCGA and GTEx datasets on the GEPIA database. Data were normalized as transcripts per kilobase million (TPM) values. TPM values were converted to log2-normalized transcripts per million [log2(TPM + 1)]. Data were shown as the mean ± standard deviation. Statistical analyses were performed using *t*-test. Error bars represented SD. *p-value < 0.05. Similar findings were found in the proteogenomic characterization of SMPD1 in pancreatic adenocarcinoma and adjacent normal pancreas tissue from the CPTAC samples (data not shown). **B** Violin plot of acid sphingomyelinase (SMPD1) expression stratified by pancreatic cancer stage from the TCGA dataset. Values were normalized as transcripts per kilobase million (TPM) values. TPM values were converted to log2-normalized transcripts per million [log2(TPM + 1)]. Statistical analyses were performed using Fisher’s exact test. *F*-value = 7.9, Pr(> *F*) = 5.76e − 05. **C** Survival analysis by acid sphingomyelinase expression in the human protein atlas database. RNA sequence data were from the open-source Human Protein Atlas (proteinatlas.org); *n* = 172 patients. All stage 4 patients were eliminated from the cohort. Median overall survival was not reached in the high expression cohort compared to 19.7 months in the SMPD1 low expression group (*p* < 0.001). Overall survival was evaluated with Kaplan–Meier survival curves with comparison made between the two groups by log-rank test

To confirm these data, we analyzed data from the Human Protein Atlas (proteinatlas.org) and the publicly available GEPIA (Gene Expression Profiling Interactive Analysis) database. We compared mRNA expression levels of SMPD1 in both tumor (TCGA) and normal tissue samples (from TCGA + Gtex). The studies revealed a higher overall expression of the enzyme in tumor samples with respect to normal tissues (Fig. 2A). This observation was confirmed also in a separate independent dataset, CPTAC, that contains pancreatic ductal adenocarcinoma tissue from 137 patients and 74 normal adjacent tissues. We also evaluated the expression levels of SPMD1 in pancreatic adenocarcinoma stratified by stage on the basis of the TCGA dataset, in order to take into consideration cancer aggressiveness. Interestingly, SPMD1 expression level was found to be higher in stage 1 but similarly elevated in more advanced stages of pancreatic ductal adenocarcinoma tumor stage (*F*-value = 7.9; Pr(> *F*) = 5,76e − 05) (Fig. 2B).

These results justify further analysis of the acid sphingomyelinase expression in tumor specimen, and thus, we correlated acid sphingomyelinase expression, based on RNA sequence data (Human Protein Atlas dataset), from 172 patients with PDAC with the long-term survival of the patients. Overall median expression of SMPD1 was 17.62 FPKM. Optimal expression cutoff value based on survival analysis was identified as 22.99. Patient details including age and stage are listed in Table 3.

**Table 3:** Human Protein Atlas Pancreatic Ductal Adenocarcinoma Cohort. Patient and staging data were obtained and analyzed from the Human Protein Atlas (preoteinatlas.org). Continuous variables compared with student *t*-test. Categorical variables were compared with Fisher’s exact and Pearson’s chi-squared tests

	**SMPD1 low** **(*n*** = **130)**	**SMPD1 High** **(*n*** = **42)**	***p*-value**
	75.6%	24.4%	
**Age (mean, SEM)**	65.2 + / − 1.0	63.0 + / − 1.7	0.25 (Student’s *t*-test)
**Sex**			0.7 (Fisher’s exact)
** Male**	56.2% (*n* = 73)	52.4% (*n* = 22)	
** Female**	43.8% (*n* = 57)	47.6% (*n* = 20)	
**Race**			0.5 (Pearson’s chi sq)
** White**	88.5% (*n* = 115)	85.7% (*n* = 36)	
** Black**	2.3% (*n* = 3)	7.1% (*n* = 3)	
** Asian**	6.9% (*n* = 9)	4.8% (*n* = 2)	
** Other, unknown**	2.3% (*n* = 3)	2.4% (*n* = 1)	
**Tumor stage**			0.001 (Pearson’s chi sq)
** I**	6.2% (*n* = 8)	31.0% (*n* = 13)	
** IIA**	20% (*n* = 26)	4.8% (*n* = 2)	
** IIB**	72.3% (*n* = 94)	54.8% (*n* = 23)	
** III**	1.5% (*n* = 2)	2.4% (*n* = 1)	
** Unknown**	0	7.1% (*n* = 3)	

The results confirm the data obtained with our cohort and show a strong correlation between acid sphingomyelinase expression in the tumor and long-term survival of the patients (Fig. 2C) Similar survival results were obtained even when all stage 1 patients were excluded from the survival analysis (not shown).

Collectively, our studies indicate that expression of the acid sphingomyelinase in PDAC strongly correlates with the prognosis and long-term survival of the patients. Low expression of the acid sphingomyelinase correlates with a poor prognosis of PDAC patients.

**Fig. 3:**
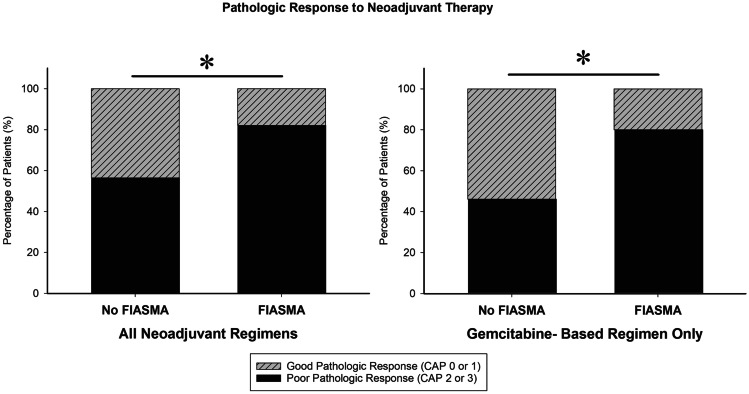
Functional inhibitors of acid sphingomyelinase during neoadjuvant treatment of PDAC impact prognosis of patients. 94 consecutive patients treated with neoadjuvant therapy followed by surgery at the University of Cincinnati were examined. Patients were categorized by the use of functional inhibitors of acid sphingomyelinase (FIASMA) during the neoadjuvant treatment period and were assessed for pathologic response to treatment on the resection specimens. Pathologic response in surgical specimens was graded based on the proportion of viable tumor according to the College of American Pathologists (CAP) grading system. Grades 0 (complete histologic response, no viable cancer cells) and 1 (near complete response, single cells or rare small groups of cancer cells) specimens were categorized as a good pathologic response, while grades 2 (partial response with residual cancer) and 3 (poor or no response with extensive residual cancer) were categorized as a poor pathologic response. A good pathologic response (CAP score of 0 or 1) was seen in 43.5% of patients not on a functional inhibitor of the acid sphingomyelinase compared to 17.9% of patients on a functional inhibitor of the acid sphingomyelinase during neoadjuvant therapy. Similar findings were seen in the subset of patients that were treated with gemcitabine-based chemotherapy (good pathologic response seen in 54% of patients not on a functional inhibitor of acid sphingomyelinase compared to 20% of patients on a functional inhibitor of acid sphingomyelinase). *p < 0.05. Pathologic responses were compared between cohorts with Fisher’s exact test

We and others have previously shown that antidepressants and other medications inhibit the acid sphingomyelinase by displacing the enzyme from lysosomal membranes resulting in the degradation of the acid sphingomyelinase within lysosomes [20–24]. This raises the question whether the application of antidepressants or other functional inhibitors of acid sphingomyelinase impacts the prognosis of patients with PDAC. We analyzed a consecutive patient cohort with PDAC treated with neoadjuvant therapy followed by surgery at the University of Cincinnati. The patients were categorized by the use of functional inhibitors of ASM during the neoadjuvant treatment period. We then assessed the response to treatment by analyzing resection specimens. A good pathologic response was defined by a CAP score of 0 or 1 and was seen in 43.5% of patients not on co-medication with a functional inhibitor of the acid sphingomyelinase compared to 17.9% of patients on a functional inhibitor of the acid sphingomyelinase co-medication during neoadjuvant therapy (Fig. 3), suggesting that inhibition of the acid sphingomyelinase inhibits the tumor response to neoadjuvant treatment.

**Fig. 4:**
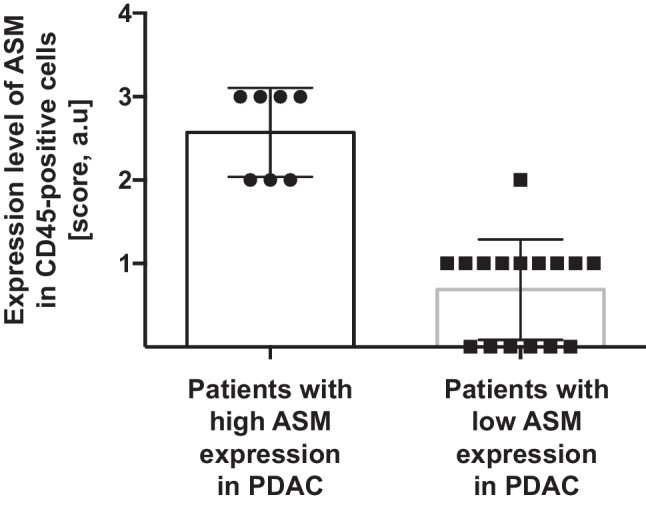
Acid sphingomyelinase expression in leukocytes in human pancreatic ductal adenocarcinoma. Paraffin sections from tumors analyzed in Fig. 2 were co-stained with FITC-labeled anti-acid sphingomyelinase and Cy3-coupled anti-CD45 antibodies. Expression of the acid sphingomyelinase was rated again between 0 and 3 + . Shown are the mean ± SD of immunofluorescence stainings in the patient groups with high and low expression of the acid sphingomyelinase in malignant PDAC, *p < 0.001, *t*-test

We also investigated expression of the acid sphingomyelinase in leukocytes of the tumor microenvironment by co-staining tumor specimen with anti-CD45 and anti-acid sphingomyelinase antibodies. These studies revealed that the expression levels of the acid sphingomyelinase in host cells correlate very similarly with acid sphingomyelinase in malignant tumor cells and with the prognosis of the patients (Fig. 4).

**Fig. 5:**
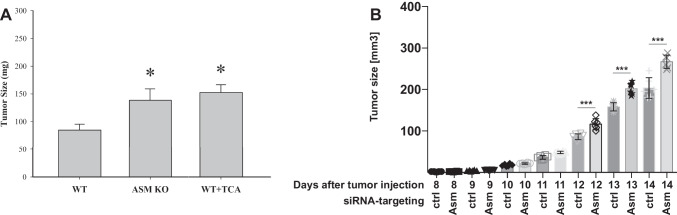
Growth of PDAC in murine models of orthotopic pancreatic ductal adenocarcinoma. **A** Wild-type mice were injected orthotopically into the pancreas with 10^6^ Pan02 pancreas cancer cells and either left untreated or treated with amitriptyline at 10 mg/kg every other day via IP injection starting at day 4 after tumor injection. In addition, we injected acid sphingomyelinase-deficient littermates with Pan02 pancreas cancer cells orthotopically into the pancreas. Tumor size was determined 15 days after injection. Shown are the mean ± SD of the tumor size from each *n* = 4–5 mice per group; *p < 0.05, ANOVA with Tukey’s post hoc test. **B** Acid sphingomyelinase expression was downregulated in KPC cells by stable transfection of siRNA targeting the acid sphingomyelinase. Controls were transfected with irrelevant siRNA. Cells were then injected into the flank of wild-type mice and tumor size was determined daily using a caliper. Shown are the mean ± SD of the tumor size from each *n* = 6 mice per group; ***p < 0.001, ANOVA with Tukey’s post hoc test

To further prove the notion that a downregulation of the acid sphingomyelinase in the tumor tissue regulates tumor progression and to discriminate the role of the acid sphingomyelinase in malignant vs. non-malignant tumor cells in PDAC progression, we established orthotopic pancreas cancers in wild-type mice and in acid sphingomyelinase-deficient mice. The wild-type mice were randomly divided into 2 groups treated with antidepressant (amitriptyline 10 mg/kg, i.p. injected every 2nd day) or vehicle, i.e., 0.9% NaCl. The size of the pancreas cancer was determined 15 days after tumor initiation. The results show that PDAC grows much faster in mice lacking the acid sphingomyelinase compared to wild-type mice (Fig. 5A). Even more importantly, the treatment of wild-type mice with antidepressants at doses that result in therapeutic blood levels also resulted in increased tumor growth (Fig. 5A).

To test whether downregulation of acid sphingomyelinase expression in PDAC also influences tumor growth in vivo, we downregulated acid sphingomyelinase expression in KPC cells by stable transfection of siRNA targeting the acid sphingomyelinase. Downregulation was confirmed by measuring the activity of the acid sphingomyelinase in cell lysates (not shown). These cells were then injected into the flank of wild-type mice, because this model allows close follow-up of the tumor size. The data indicate that PDAC lacking acid sphingomyelinase showed significantly larger tumors than wild-type tumor cells (revised Fig. 5B).

**Fig. 6:**
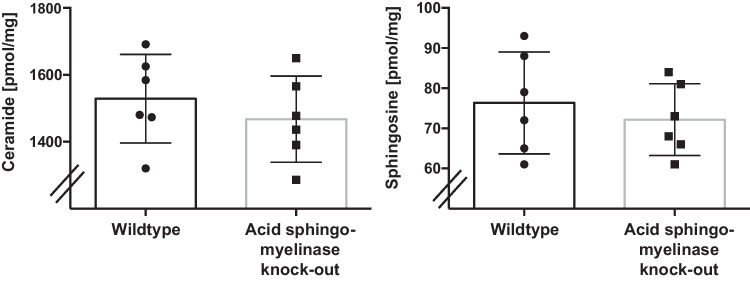
Ceramide and sphingosine in PDAC. KPC PDAC cells were injected into the flank of wild-type and acid sphingomyelinase-deficient mice, the tumors were allowed to grow for 10 days, removed, homogenized and ceramide **A **and sphingosine **B **levels were determined biochemically. Shown are the mean ± SD from each *n* = 6 mice per group; *p < 0.05, *t*-test

Next, we determined ceramide and sphingosine in KPC PDAC cells injected into the flank of wild-type and acid sphingomyelinase-deficient mice (Fig. 6A, B). However, the results do not show any significant differences in total lysates of the tumors (Fig. 6A, B).

## Discussion

Our data indicate an important role of the acid sphingomyelinase for progression of PDAC and therapy of patients with PDAC. The data obtained from two independent tissue banks and patient groups indicate that a high expression of the acid sphingomyelinase in the tumor tissue strongly correlates with long-term survival of PDAC patients. These data are based, at least for the patient group from the University of Cincinnati, on tissue samples from untreated patients. These patients were on a surgery-first approach and the malignant tumor tissue was removed prior to any chemotherapy, indicating that expression of the acid sphingomyelinase in the tumor tissue is a true prognostic marker for PDAC. These data suggest that the acid sphingomyelinase could serve as a novel marker to predict tumor progression in patients with PDAC. The acid sphingomyelinase may also serve as marker to determine whether a patient requires more or less aggressive treatment. However, this would need to be investigated in randomized clinical trials.

Thus, in summary, the present paper is a clinically applied paper that identifies with 2 independent data bases a highly interesting link between expression of the acid sphingomyelinase and prognosis in PDAC. Molecular mechanisms of how the acid sphingomyelinase mediates the resistance to PDAC are beyond the present manuscript and need to be defined in detailed future studies.

The patient data were obtained from staining of tumor specimen and analysis of the acid sphingomyelinase in the malignant tumor cells. The other data sets were obtained from the Human Protein Atlas and was based on biopsies. It reflects mRNA expression of the acid sphingomyelinase in these samples without specification of any cell type. Our data obtained in acid sphingomyelinase-deficient mice that were injected with Pan02 pancreas carcinoma cells suggests that not only the expression of the acid sphingomyelinase in tumor cells, but also the expression of the acid sphingomyelinase in host cells is mostly important for tumor progression, since in our orthotopic tumor model in knock-out mice, the acid sphingomyelinase is deficient in host cells, but not in the malignant tumor cells. However, our data may also allow the conclusion that a high expression of the acid sphingomyelinase in malignant PDAC alters tumor-released factors that mediate immune alterations of host cells, which also depend on the expression level of the acid sphingomyelinase.

Thus, future studies must analyze the expression of the acid sphingomyelinase in the entire tumor including all types of host cells, blood leukocytes, local lymph nodes, the bone marrow, and, if possible, also the spleen, as well as of the secretory acid sphingomyelinase in the blood plasma, to define which cell type(s) has a high expression of the acid sphingomyelinase and may control the present phenotype.

Our data indicate that inhibition of the acid sphingomyelinase in PDAC patients may result in faster tumor growth and/or reduced response to chemotherapy. Tricyclic antidepressants and selective serotonin reuptake inhibitors (SSRI) inhibit the acid sphingomyelinase. These antidepressants induce the release of acid sphingomyelinase from lysosomal membranes, thereby triggering the degradation of the enzyme in the lysosomal lumen [20–24]. In detail, the acid sphingomyelinase seems to predominantly associate with intralysosomal membranes and the interaction of the enzyme with these membranes is targeted by drugs such as antidepressants [20, 22]. Antidepressants inhibiting the acid sphingomyelinase are weak bases that are protonated in lysosomes and thereby trapped in lysosomes. The organic ring system of these compounds may bind to lipid membranes, whereas the protonated tertiary amine displaces acid sphingomyelinase from lysosomal membranes and thereby induces degradation of the acid sphingomyelinase. Thus, these weak bases do not directly inhibit acid sphingomyelinase activity but rather functionally inhibit the enzyme.

Our data indicate that inhibition of the acid sphingomyelinase in patients treated with tricyclic antidepressants or SSRI results in increased tumor progression and a reduced response to neoadjuvant treatment. These data are clinically very important and suggest that patients with PDAC should not be treated with FIASMA antidepressants or other compounds that inhibit the acid sphingomyelinase.

In summary, our data show that expression of the acid sphingomyelinase correlates with long-term survival of pancreas cancer patients. The data suggest the acid sphingomyelinase as a novel marker for pancreas cancer prognosis. They also indicate that patients with pancreas cancer should not be treated with pharmacological inhibitors of the acid sphingomyelinase, such as many antidepressants. Finally, our data suggest that treatment of patients with PDAC with recombinant acid sphingomyelinase to increase endogenous levels of acid sphingomyelinase expression may serve to develop novel treatments of PDAC.

## Methods

### Patient cohorts

The use and collection of all clinical and pathologic data and specimens were performed with approval and in accordance with the guidelines established by the University of Cincinnati Institutional Review Board (IRB 2019–0324).

#### University of cincinnati medical center cohort surgery-first cohort

Consecutive patients (*n* = 23) with resectable pancreatic ductal adenocarcinoma (PDAC) undergoing a surgery-first approach from 2014–2019 with available paraffin-embedded tissue blocks were included for analysis. Only patients undergoing a surgery first approach were included for analysis in order to evaluate inherent baseline expression of acid sphingomyelinase within the tumor. Patient charts were reviewed and patient demographic, clinical, operative, and pathologic data were collected.

#### Human protein atlas cohort

The human protein atlas (proteinatlas.org) is a publicly available data source licensed under the Creative Commons Attribution-ShareAlike 3.0 International License that includes a pathologic atlas of the human cancer transcriptome [25]. The database was queried for mRNA expression of the sphingomyelin phosphodiesterase 1 (SMPD1) gene in patients with pancreatic ductal adenocarcinoma. SMPD1 expression levels and clinical characteristics of 172 patients were collected which included survival and cancer stage. Stage 4 patients were excluded from the cohort for analysis.

#### The cancer genome atlas and clinical proteomic tumor analysis consortium cohort

The Cancer Genome Atlas (TCGA) and Genotype-tissue expression (GTEx) TCGA is a freely web-based accessible database, which collects NGS data from more than 10,000 tumors across 33 cancer types until 2018 [26]. Gene expression and clinical data were taken into consideration in the present study. Genotype-tissue expression (GTEx) GTEx provides publicly available gene expression data from 53 normal tissue sites across nearly 1000 people by RNA sequencing [27]. The Clinical Proteomic Tumor Analysis Consortium (CPTAC) dataset, publicly available since 2021, is based on whole-genome sequencing (WGS) and whole-exome sequencing (WES) of 140 pancreatic cancers with 67 normal adjacent tissues giving rise to proteogenomic characterization of pancreatic ductal adenocarcinoma [28]. The cBioPortal for Cancer Genomics (http://www.cbioportal.org), GEPIA (gene expression profiling interactive analysis) (http://gepia.cancerpku.cn/), and the UALCAN database (http://ualcan.path.uab.edu) analysis tools were employed.

#### University of cincinnati neoadjuvant cohort

Consecutive patients (*n* = 94) with biopsy-proven pancreatic ductal adenocarcinoma that underwent neoadjuvant chemotherapy followed by surgical resection from 2012–2022 were included for analysis. Clinicopathologic data were collected from our institutional database. Medical reconciliation records were reviewed prior to, during, and at the completion of neoadjuvant chemotherapy for all medications. Patients were then categorized based on the use of functional inhibitors of acid sphingomyelinase (FIASM) during treatment [20, 29]. Pathologic response in surgical specimens was graded based on the proportion of viable tumor according to the College of American Pathologists grading system. Grades 0 (complete histologic response, no viable cancer cells) and 1 (near complete response, single cells or rare small groups of cancer cells) specimens were categorized as a good pathologic response while grades 2 (partial response with residual cancer) and 3 (poor or no response with extensive residual cancer) were categorized as a poor pathologic response.

### Immunohistochemical Staining

Acid sphingomyelinase staining protocol: Tissue slides were deparaffinized and rehydrated in xylene substitute and gradients of ethanol after heating for 1 h at 65 °C. Permeabilization was done with 1% Triton X-100 for 15 min on a shaker plate and then washed with PBS-Tween buffer. Antigen retrieval was done with Tris–EDTA, pH 9.0 buffer by using Thermo-Electron Corporation Shandon Tissue Wave 2, HIER1 program. Slides were cooled to room temperature, washed with PBS-Tween buffer, and then treated with 3% H_2_O_2_ for 1 h at 37 °C to block endogenous peroxidase. Specimens were then incubated with rabbit primary antibody, anti-Smpd1 (Proteintech, Cat# 14,609–1-AP) at 1:200 dilution at 4 °C overnight. The sections were extensively washed with PBS-Tween buffer and then incubated with biotinylated goat anti-rabbit secondary antibody at room temperature for 30 min. The specimens were washed with PBS-Tween buffer and then incubated with Streptavidin-HRP pre-diluted (SAV-HRP, # BD550946, ready to use) for 30 min at room temperature. The specimens were then treated with diaminobenzidine DAB (HRP substrate, ThermoScientific, TA-125-QHDX) for about 1 min and the reaction stopped by transfer to distilled water. The specimens were then counterstained with hematoxylin. Sections were mounted onto slides with Permount (SP15-500, Thermo Fisher Scientific, USA) for review.

### Evaluation of immunohistochemical staining in pathologic specimens

All slides were reviewed and scored by a pathologist (JW) blinded to the identity of the samples. The pathologist is an expert in pancreatic cancer. Only staining within the invasive tumor component was considered when grading the staining. The slides were then scored on an ordinal scale based on the intensity of acid sphingomyelinase staining: 0 = negative or no staining, 1 +  = weak staining, 2 +  = intermediate/moderate staining, and 3 +  = strong staining intensity. Patients were then categorized as ASM low expression (staining intensity of 0 or 1 +) or ASM high expression (staining intensity of 2 + or 3 +) for comparison.

### Orthotopic pancreatic cancer murine model

All animal experiments were approved by the University of Cincinnati Ethic Committee and the Institutional Animal Care and Use Committee. Eight-week-old, wild-type male, C57BL/6 J mice were purchased from Jackson Labs (000,664, Jackson Labs, USA). Acid sphingomyelinase-deficient mice (*Smpd1*^−*/*−^) were bred and genotyped at the animal facility of the University of Cincinnati. We used young acid sphingomyelinase-deficient mice (maximum age 10 weeks) to avoid sphingomyelin accumulation. Mice were anesthetized using 120 mg/kg ketamine plus 20 mg/kg xylazine. Orthotopic injection was performed as described by Tepal et al. [30]. In detail, a left subcostal incision was made just below the rib cage and the pancreas was identified. The tumor cell suspension was created by mixing 25 µL of Matrigel with 25 µL of Pan02 cells (National Cancer Institute-Frederick Cancer Research and Development Center, Frederick, MD, USA) containing 1 × 10^6^ cells. Pan02 cells were cultured in DMEM + 10% FBS medium, under 37 ℃ and 5% CO_2_; no antibiotic was added. Cells were washed, trypsinized, and washed in PBS at least 3 times prior to injection. The tumor suspension was slowly injected into the pancreas and the needle left in place for 60 s to allow the Matrigel to set. After ensuring hemostasis, the abdomen was closed in 2 layers using 3–0 silk suture. Wild-type mice were then randomly divided into 2 groups. One group was treated with 10 mg/kg of the antidepressant and functional inhibitor of the acid sphingomyelinase amitriptyline or vehicle, i.e., 0.9% NaCl. Amitriptyline or 0.9% NaCl was injected into the peritoneal cavity every other day. The size of the pancreas cancer was determined in all groups 15 days after tumor initiation.

### Flank tumor model

A total of 25,000 KPC tumor cells were injected subcutaneously into the flank of wild-type or acid sphingomyelinase-deficient C57BL/6 mice. Tumor growth was monitored daily using calipers. Mice were sacrificed at day 14 after tumor cell injection; the tumors were removed and shock frozen in liquid nitrogen for further analysis.

### Transfection of siRNA targeting acid sphingomyelinase

KPC (20 × 10^6^ cells) were incubated with commercial siRNA targeting acid sphingomyelinase (Santa Cruz Inc., # sc-41651-SH) or a control construct for 15 min on ice. Cells were then electroporated at 500 V, 5 impulses with each 3-ms duration, left on ice for 15 mice, transferred into 10 mL MEM supplemented with 10 mM HEPES (pH 7.4; Carl Roth GmbH, Karlsruhe, Germany), 2 mM l-glutamine, 1 mM sodium pyruvate, 100 μM nonessential amino acids, 100 U/mL penicillin, 100 μg/mL streptomycin (all from Invitrogen), and 10% FCS (PAA Laboratories GmbH, Coelbe, Germany) and grown for 2 days. Dead cells were removed and positive cells were selected using 3.5 μg/mL puromycin. Stable transfected cells were used as bulk cultures. The activity of the acid sphingomyelinase in transfected cells was determined using a biochemical assay. To this end, cells were lysed in 250 mM sodium acetate (pH 5.0) and 0.2% NP40 for 5 min, removed from the plate and 270 μL of the lysates were incubated with 30 μL of 0.05 μCi [^14^C]sphingomyelin (52 mCi/mmol; ARC). Prior to addition, the substrate [^14^C]sphingomyelin was dried for 10 min in a SpeedVac, resuspended in 250 mM sodium acetate (pH 5.0) and 0.1% NP40 and sonicated for 10 min in a bath sonicator. The samples were incubated for 30 min at 37 °C with shaking at 300 rpm. The enzymatic reaction was terminated by extraction in 4 volumes of CHCl_3_:CH_3_OH (2:1, v/v), the samples were centrifuged, and an aliquot of the upper aqueous phase was scintillation-counted to determine the release of [^14^C]phosphorylcholine from [^14^C]sphingomyelin.

### Immunohistochemistry of tumor sections for acid sphingomyelinase and CD45

Tumor tissue sections were prepared as above, dewaxed, and rehydrated. To retrieve the antigens, sections were treated with pepsin (Digest All; #003,009, Invitrogen) for 30 min at 37 °C, washed and blocked for 10 min at room temperature with PBS, supplemented with 5% fetal calf serum (FCS). Samples were washed once in PBS and stained with polyclonal anti-human acid sphingomyelinase (1:100 dilution in PBS + 1% FCS, #AF5348, R&D Systems) for 45 min, washed 3 times in PBS plus 0.05% Tween 20 and once with PBS. Samples were then stained with FITC-F(ab)_2_ fragments against goat IgG (1:500 dilution, Jackson Immunoresearch, #705–096-147) for 30 min in PBS + 1% FCS and washed again 3 times in PBS plus 0.05% Tween 20 and once with PBS. Finally, the samples were stained with PE-coupled mouse anti-human CD45-antibodies (clone 2D1; 1:500 dilution, R&D Systems, FAB1430P) for 30 min in PBS + 1% FCS. Specimens were washed again 3 times in PBS plus 0.05% Tween 20 and embedded in Mowiol. Samples were evaluated by confocal microscopy on a Leica TCS-SL confocal microscope equipped with a 40 × lens, and images were analyzed with Leica LCS software (Leica Microsystems, Mannheim, Germany). All comparative samples were measured at identical settings. Control stainings were performed with FITC-labeled secondary antibodies only or with irrelevant goat IgG followed by staining FITC-labeled FITC-F(ab)_2_ anti-goat IgG. These controls revealed very weak stainings and confirmed the specificity of the antibody stainings. Fluorescence intensities were quantified using Image J.

### Ceramide and sphingosine measurements in isolated pancreatic ductal adenocarcinoma

Tumors were isolated, homogenized in H_2_0 and extracted in CHCl_3_/CH_3_OH/1N HCl (100:200:1, v/v/v). The lower phase was dried and resuspended in a detergent solution consisting of 7.5% (w/v) n-octyl glucopyranoside, 5 mM cardiolipin in 1 mM diethylenetriamine-pentaacetic acid. The kinase reaction was initiated by addition of 0.001 units sphingosine kinase in 50 mM HEPES (pH 7.4), 250 mM NaCl, 30 mM MgCl_2_ 10 μM ATP and 10 μCi [^32^P]γATP or 10 μL diacylglycerol (DAG) kinase (GE Healthcare Europe, Munich, Germany), 0.1 M imidazole/HCl (pH 6.6), 0.2 mM DTPA, 70 mM NaCl, 17 mM MgCl_2_, 1.4 mM ethylene glycol tetraacetic acid, 2 mM dithiothreitol, 1 μM adenosine triphosphate (ATP), and 5 μCi [^32^P]γATP. Samples were incubated for 30 min at 37 °C with shaking (350 rpm) and the kinase reaction was terminated by extraction of lipids. The sphingosine kinase reaction was terminated by addition of 20 μL 1N HCl, 800 μL CHCl_3_:CH_3_OH:1N HCl (100:200:1, v/v/v), and 240 μL each of CHCl_3_ and 2 M KCl. Phases were separated; the lower phase was collected, dried, dissolved in 20 μL CHCl_3_:CH_3_OH (1:1, v/v), and separated on Silica G60 TLC plates with CHCl_3_:CH_3_OH:acetic acid:H_2_O (90:90:15:5, v/v/v/v) as developing solvent. The ceramide kinase reaction was terminated by extraction with 1000 μL CHCl_3_:CH_3_OH:1N HCl (100:100:1, v/v/v), 170 μL buffered saline solution (135 mM NaCl, 1.5 mM CaCl_2_, 0.5 mM MgCl_2_, 5.6 mM glucose, 10 mM HEPES; pH 7.2) and 30 μL of 100 mM EDTA, lipids were extracted, separated by Silica G60 TLC plates with chloroform/acetone/ methanol/acetic acid/H_2_O (50:20:15:10:5, v/v/v/v/v), as developing solvent. The TLC plates were analyzed with a phosphorimager. Sphingosine and ceramide levels were determined with a standard curve of C18-sphingosine or C16 and C24 ceramide.

### Statistics

Clinicopathologic data were obtained from the electronic medical records and our institutional prospectively maintained pancreatic cancer database. Variables include patient age, gender, histologic grade, lymphovascular invasion, perineural invasion, stage, lymph node status, and oncologic status. Statistical analyses were performed using JMP Pro v15 (SAS, Cary, NC) and SigmaPlot 14.5 (Systat Software, Inc., Palo Alto, CA). Data were classified as categorical and continuous variables. Categorical variables were described with counts and percent with comparison made with Pearson’s chi-squared and Fisher’s exact test, as appropriate. Continuous variables were examined using Student’s *t*-test. Survival analyses were performed using Kaplan–Meier methodology with log-rank test for group comparison. *p*-values < 0.05 were considered statistically significant.

## Data Availability

All data are presented in the manuscript. All material is freely available.
